# Enhanced CO_2_ Capture through SAPO-34 Impregnated with Ionic Liquid

**DOI:** 10.1021/acs.langmuir.4c00466

**Published:** 2024-04-19

**Authors:** Nannan Ye, Yusi Shen, Yifeng Chen, Jian Cao, Xiaohua Lu, Xiaoyan Ji

**Affiliations:** † State Key Laboratory of Materials-Oriented Chemical Engineering, College of Chemical Engineering, Nanjing Tech University, Nanjing 210009, P. R. China; ‡ CAF, Key and Open Laboratory of Forest Chemical Engineering, Key Laboratory of Biomass Energy and Material, SFA, National Engineering Laboratory for Biomass Chemical Utilization, Institute of Chemical Industry of Forest Products, Nanjing 210042, P. R. China; § Suzhou Laboratory, Suzhou 215100, P. R. China; ∥ Division of Energy Science/Energy Engineering, Luleå University of Technology, Luleå 97187, Sweden

## Abstract

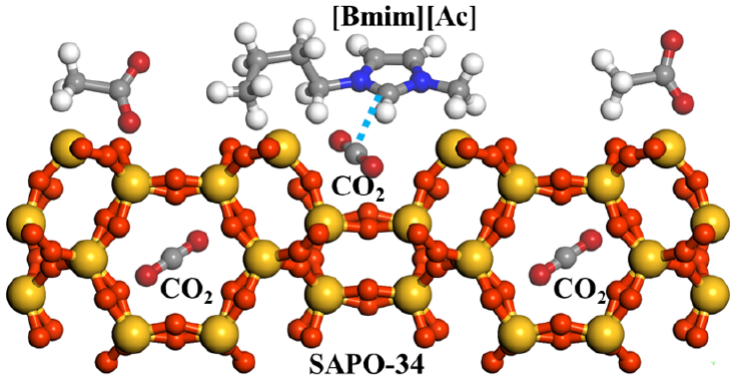

The concurrent utilization of an adsorbent and absorbent for carbon dioxide (CO_2_) adsorption with synergistic effects presents a promising technique for CO_2_ capture. Here, 1-butyl-3-methylimidazole acetate ([Bmim][Ac]), with a high affinity for CO_2_, and the molecular sieve SAPO-34 were selected. The impregnation method was used to composite the hybrid samples of [Bmim][Ac]/SAPO-34, and the pore structure and surface property of prepared samples were characterized. The quantity and kinetics of the sorbed CO_2_ for loaded samples were measured using thermogravimetric analysis. The study revealed that SAPO-34 could retain its pristine structure after [Bmim][Ac] loading. The CO_2_ uptake of the loaded sample was 1.879 mmol g^–1^ at 303 K and 1 bar, exhibiting a 20.6% rise compared to that of the pristine SAPO-34 recording 1.558 mmol g^–1^. The CO_2_ uptake kinetics of the loaded samples were also accelerated, and the apparent mass transfer resistance for CO_2_ sorption was significantly reduced by 11.2% compared with that of the pure [Bmim][Ac]. The differential scanning calorimetry method revealed that the loaded sample had a lower CO_2_ desorption heat than that of the pure [Bmim][Ac], and the CO_2_ desorption heat of the loaded samples was between 30.6 and 40.8 kJ mol^–1^. The samples exhibited good cyclic stability. This material displays great potential for CO_2_ capture applications, facilitating the reduction of greenhouse gas emissions.

## Introduction

Carbon dioxide (CO_2_) emission is a major challenge to global environmental degradation and warming, and efficient CO_2_ capture is of great industrial, economic, and environmental significance.^1−3^ In this case, it is urgent to design new materials for CO_2_ capture.^4^ Currently, different materials have been developed to capture CO_2_, including absorbents, adsorbents, and absorbents combined with adsorbents.^5^ Among them, the composite of absorbents and adsorbents (i.e., absorbents combined with adsorbents) combines the advantages of both absorbent and adsorbent, and as reported, they can significantly improve the CO_2_ sorption capacity and selectivity, as well as the sorption rate.^6,7^ Currently, amines or ionic liquids (ILs) combined with solid adsorbents are common composites developed for CO_2_ capture. Studies have shown that such composites can enhance the sorption capacity, rate, and selectivity of CO_2_.^7−9^ However, these materials commonly exhibit a trade-off of sorption amount and desorption heat. For example, the PEI/SiO_2_ sorbent exhibited a CO_2_ sorption of more than 1.39 mmol g^–1^ at 313 K, but the desorption heat was 100 kJ mol^–1^.^10^ This can be attributed to the strong chemical interaction between the amine functional group and CO_2_. Instead, when the activated carbon (AC) was loaded with (vinylbenzyl)trimethylammonium glycine ([Vbtma][gly]), AC-20 wt % [Vbtma][gly], the CO_2_ sorption capacity and desorption heat at 1.0 bar and 313 K were 0.82 mmol g^–1^ and 13.00 kJ mol^–1^, respectively.^11^ The use of the AC loaded with 1-butyl-3-methylimidazole acetate ([Bmim][Ac]) resulted in an almost completely selective CO_2_ sorption with respect to N_2_ and CH_4_, while with relatively low sorption amount (0.771 mmol g^–1^) and desorption heat (27.4 kJ mol^–1^).^12^ Therefore, the selection of a suitable absorbent and adsorbent to prepare composites with high sorption capacity and low desorption energy demand is of great significance.

The performance of the absorbent–adsorbent composite depends on both constituents. For the adsorbents, the molecular sieve materials have a good stable structure, large specific surface area, and adjustable surface properties, possessing promising CO_2_ adsorption performance.^13,14^ In particular, the SAPO-34 molecular sieves with uniform pores have been reported to exhibit a high CO_2_ adsorption capacity and selectivity while also possessing a low CO_2_ desorption heat. The report states that SAPO-34 has a CO_2_ adsorption amount of up to 1.34 mmol g^–1^ and a low desorption heat of 33 kJ mol^–1^ at 303 K.^15^ Neishabori Salehi et al. also found that SAPO-34 had a CO_2_ adsorption of 1.571 mmol g^–1^ and desorption heat of 36.74 kJ mol^–1^ at 298 K,^71^ and Tamnanloo et al. reported a CO_2_ adsorption capacity of 1.497 mmol g^–1^ with a high CO_2_/CH_4_ selectivity at 303 K and 1.0 bar.^16^ Regarding the absorbents, the unique properties of ILs, such as low vapor pressure, volatility, high affinity, and solubility for CO_2_,^17,18^ make them an efficient and environmentally suitable candidate for CO_2_ capture.^19−21^ In particular, the ILs containing acetate-based anions have better absorption properties for CO_2_, e.g., [Bmim][Ac] exhibits a high affinity for CO_2_^22,23^ and thus desirable CO_2_ uptake at 298 K up to 2.19 mmol g^–1^. According to the density functional theory prediction, the interaction energy between [Bmim][Ac] and CO_2_ was 36.37 kJ mol^–1^, indicating slow energy usage for desorption.^24^

Here, we combined a molecular sieve adsorbent with an IL absorbent to enhance the CO_2_ capture performance. Specifically, the impregnation method was used to load [Bmim][Ac] onto SAPO-34 that is thermally and chemically stable and has a high CO_2_ adsorption performance. We characterized the prepared samples using Brunauer–Emmett–Teller (BET), X-ray diffraction (XRD), Fourier transform infrared (FTIR), scanning electron microscopy (SEM), energy dispersive X-ray spectroscopy (EDX), X-ray photoelectron spectroscopy (XPS), thermogravimetric analysis (TGA), and differential scanning calorimetry (DSC). The study investigated the influence of IL loading amount (2.84, 5.40, 9.97, and 15.38 wt %), sorption temperatures (30, 40, and 50 °C), and desorption temperatures (30, 40, 50, and 60 °C) on the sorption capacity, kinetics, selectivity, and the CO_2_ desorption heat as well as cyclic stability.

## Materials and Methods

### Materials

[Bmim][Ac] (CAS NO. 284049-75-8, > 98 wt %) was purchased from Lanzhou Institute of Chemical Physics, Chinese Academy of Sciences. Prior to use, IL was dried in a vacuum drying oven at 353 K for 72 h and then stored in a desiccator filled with silica gel. SAPO-34 (CAS NO. 9893-000-2, Si/Al ratio = 0.25) was purchased from Nankai University Catalyst Company, China, and it was calcined in a muffle furnace at 673 K for 6 h before the experiment. Potassium bromide (KCl, CAS NO. 7758-02-3, spectral grade) was supplied by Sigma-Aldrich. Methanol (CAS NO. 67-56-1, ≥ 99.7%) was purchased from Lingfeng Chemical Reagent Co., China. All gases used in the sorption–desorption equilibrium measurements, N_2_ and CO_2_, were purchased from Nanjing Special Gas Plant Co., Ltd.

### Preparation of SAPO-34-[Bmim][Ac]

**Figure 1:**
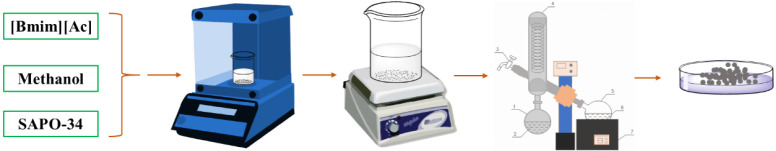
Preparation of SAPO-34-[Bmim][Ac].

The SAPO-34-IL materials were prepared using the impregnation-vacuum rotary evaporation method,^25^ as depicted in Figure 1. An amount of IL was added to the anhydrous methanol under magnetic stirring and mixed for a certain amount of time. Then, the molecular sieve SAPO-34 was added, and the stirring was continued. After that, methanol was removed through vacuum rotary evaporation at 333 K to obtain composite materials. Finally, the composite materials were dried in a vacuum drying oven at 353 K overnight to remove any remaining solvent. The prepared samples were stored in a laboratory desiccator and named SAPO-34-IL-*x* (*x* = 1, 2, 3, and 4) based on their mass ratios of IL to SAPO-34.

### Characterization of Samples

#### BET

The argon adsorption and desorption isotherms for each sample were measured at 77 K to determine the surface area, total pore volume, and average pore size through a Micromeritics Tristar II 3020 analyzer (Micromeritics, USA). Prior to the argon adsorption and analyses using the liquid nitrogen, the samples were degassed pretreatment under vacuum at 353 K for 8 h.

#### TGA and DSC

The thermal stability, the mass fraction of IL in the loaded sample, and the values of CO_2_ desorption heat for [Bmim][Ac], SAPO-34, and SAPO-34-IL were determined using TGA and DSC (TA, SDT650). The samples were heated from room temperature to 373 K at 10 K min^–1^ under 50 mL min^–1^ nitrogen (N_2_) atmosphere until no significant weight loss was observed and then heated to 673 K at 10 K min^–1^. The decomposition temperatures of pure and loaded IL, as well as the loading of IL were calculated using thermogravimetric (TG) curves and derivative TG curves. Additionally, the CO_2_ desorption heat was obtained from the DSC heat flow curve of the CO_2_ sorption.

#### FTIR

The surface functional groups of the samples were identified using FTIR (NicoletiN10) with a scanning ranging from 4000 to 400 cm^–1^ and a resolution of 4 cm^–1^. A small amount of the sample was mixed with dry potassium bromide, ground, and pressed into a transparent sheet using a mold.

#### XRD

The crystal structures of the prepared samples were analyzed using XRD spectroscopy (D8Adavance). The X-ray generator was set to 40 kV voltage and 20 mA current to produce Cu Kα_1_ (1.5406 Å) radiation. Diffraction patterns were obtained at 2θ values between 5° and 40°.

#### SEM and EDX

The morphology and size of samples were evaluated using SEM (S-4800, Hitachi). The elemental compositions of the samples were determined through EDX.

#### XPS

The surface elemental compositions of molecular sieves SAPO-34 and SAPO-34-IL were determined by XPS using a PHI 5000 Versa Probe equipped with Al Kα radiation.

### CO_2_ Sorption and Desorption

The sorption/desorption studies of CO_2_ were conducted by TGA,^26,27^ as depicted in Table 1. About 2–3 mg of the sample was placed in an alumina pan and heated from room temperature to 373 K at a rate of 10 K min^–1^ under 50 mL min^–1^ N_2_ atmosphere for 20 min to eliminate water and sorbed gases. Subsequently, the temperature was cooled to the sorption temperature, and 50 mL min^–1^ of CO_2_ was introduced for up to 150 min to achieve saturated sorption. Finally, the operation was switched to 50 mL min^–1^ N_2_ for desorption for 30 min from the sorption temperature to the desorption temperature. The sorption/desorption studies were repeated to perform 6 cycles.

**Table 1:** Details of CO_2_ Sorption and Desorption Experiments

samples	mass (mg)	loadings (wt %)	sorption *T* (K), CO_2_ flow (mL min^–1^), *t* (min)	desorption *T* (K), N_2_ flow (mL min^–1^), *t* (min)
SAPO-34	2.771	–	303, 50, 150	333, 50, 30
SAPO-34-IL-1	2.015	2.84	303, 50, 150	333, 50, 30
SAPO-34-IL-2	2.252	5.40	303, 50, 150	333, 50, 30
SAPO-34-IL-3	2.338	9.97	303, 50, 150	333, 50, 30
SAPO-34-IL-4	2.410	18.38	303, 50, 150	333, 50, 30

## Results and Discussion

### Characterization of the SAPO-34-IL Samples

To characterize the properties and structure of the prepared materials, we conducted argon adsorption–desorption isotherms, TGA, DSC, FTIR, XRD, SEM, EDS, and XPS analysis.

**Figure 2:**
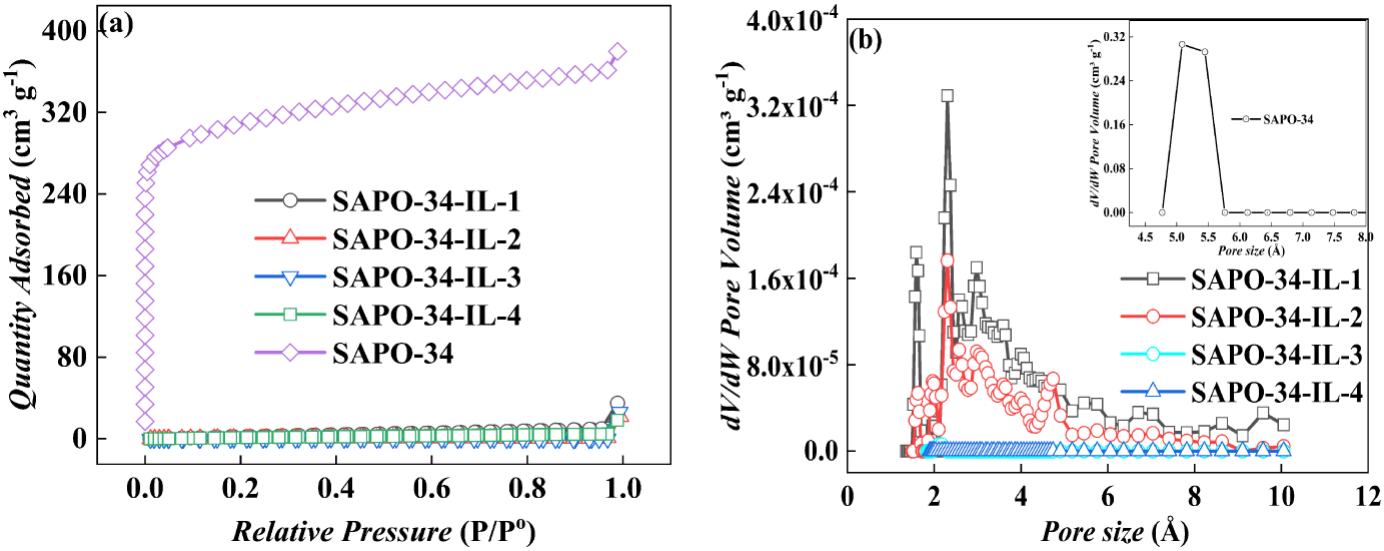
(a) Isotherm for argon sorption/desorption and (b) pore size distribution of SAPO-34 and SAPO-34-IL.

Figure 2 and Table 2 show the sorption–desorption isotherms, as well as pore volume and pore size distributions of the pristine SAPO-34 and SAPO-34-IL. As shown in Figure 2a, the isotherm of the pristine SAPO-34 exhibits a typical type I, indicating an interior pore structure dominated by micropores. The specific surface areas of the samples decrease sharply from 460 m^2^ g^–1^ for pristine SAPO-34 to 8.6 m^2^ g^–1^, 4.823 m^2^ g^–1^, 1.926 m^2^ g^–1^, and 0.477 m^2^ g^–1^, for SAPO-34-IL-1, SAPO-34-IL-2, SAPO-34-IL-3, and SAPO-34-IL-4, respectively. The corresponding pore volume also quickly decreased from 0.272 cm^3^ g^–1^ for the pristine SAPO-34 to 0.0250 cm^3^ g^–1^, 0.0180 cm^3^ g^–1^, 0.0154 cm^3^ g^–1^, and 0.0130 cm^3^ g^–1^ for SAPO-34-IL-1, SAPO-34-IL-2, SAPO-34-IL-3, and SAPO-34-IL-4, respectively, as shown in Figure 2b. This means that the IL molecules cover the pore openings of the molecular sieve, leading to a sharp decrease in both the internal specific surface area and the pore volume. As the amount of IL loading further increases, it accumulates on the surface of the molecular sieve. It is reasonable that the IL mainly covers the orifice, because the size of the IL is larger than the pore size of the molecular sieve.^28^

**Table 2:** Textural Properties of SAPO-34 and SAPO-34-IL

samples	*S*_BET_ (m^2^ g^–1^)	*V*_P_ (cm^3^ g^–1^)
SAPO-34	460	0.272
SAPO-34-IL-1	8.6	0.0250
SAPO-34-IL-2	4.823	0.0188
SAPO-34-IL-3	1.926	0.0154
SAPO-34-IL-4	0.477	0.0130

**Figure 3:**
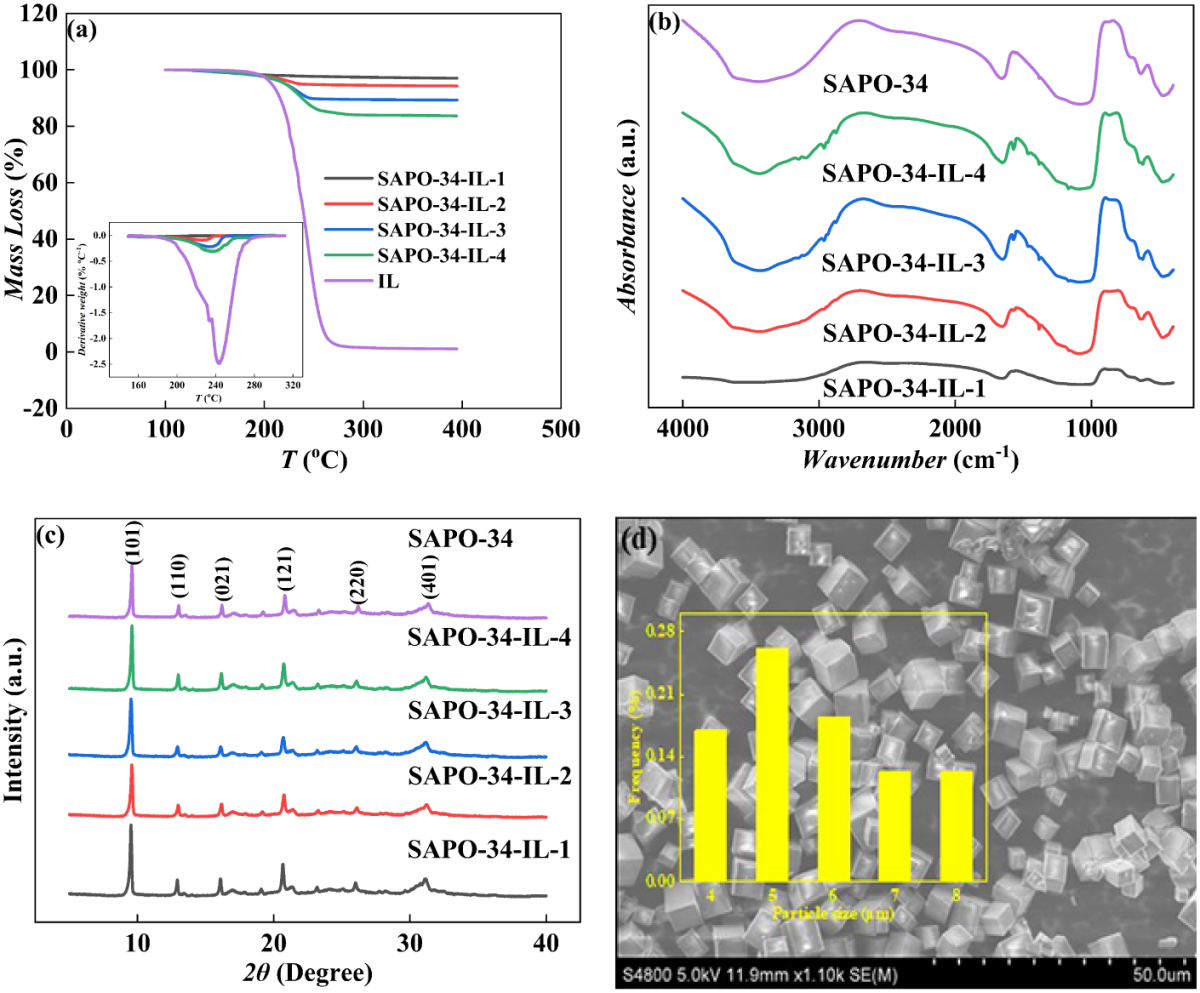
Characterization of SAPO-34 and SAPO-34 with different IL loadings: (a) TGA curves, (b) FTIR pattern, (c) XRD pattern, and (d) SEM image.

To determine the thermal stability of the loaded IL and its loading amount, TGA was conducted under the N_2_ atmosphere, as shown in Figure 3a. The decomposition curves indicate that pure [Bmim][Ac] remains stable up to 494 K and then starts to decompose rapidly. Furthermore, the thermal decomposition temperatures of the loaded ILs were measured to be 459, 478, 484, and 489 K. This suggests a slight decrease in decomposition temperatures when IL is loaded onto the surface of the molecular sieve, which is in agreement with the observations of Durak et al.^12^ According to the TGA results, the loading amounts of ILs in the composites were calculated, which were 2.84, 5.40, 9.97, and 15.38 wt %. The TG decomposition curves and derivative TG curves of the samples indicated a single-step decomposition for both the pure IL and the composites.

Figure 3b shows the surface functional groups of the samples using FTIR spectroscopy. The characteristic peak at 475 cm^–1^ represents the bending vibration of the tetrahedron of SiO_4_. The hexagonal ring of SAPO-34 shows a characteristic peak at 638 cm^–1^. The peaks at wavenumbers of 1081 and 1654 cm^–1^ are attributed to O–P–O asymmetric vibrational peaks of the asymmetric water and physical adsorbed water, respectively. The peak at a wavenumber of 3435 cm^–1^ is attributed to the bridging hydroxyl group.^29,30^ When IL is loaded onto SAPO-34, the composite materials exhibit the characteristic peaks of IL, where the characteristic peaks at 863 and 1168 cm^–1^ are attributed to the C–N stretching vibration and imidazole cation,^31^ and those at 1383 and 1571 cm^–1^ correspond to the characteristic peaks of C–O and C= O, respectively.^32^ The characteristic peaks of the methyl functional group −CH_3_ appear as asymmetric stretching vibration and symmetric stretching vibration at 2875 cm^–1^ and 2962 cm^–1^.^24^ The C–H stretching vibration and N–H stretching vibration of the aryl ring have two new characteristic peaks at 3102 cm^–1^ and 3148 cm^–1^.^24^ Therefore, the surface of the SAPO-34 molecular sieve was successfully loaded with IL.

To evaluate the effect of IL loading on the crystal structure of the molecular sieves, XRD was performed on the samples before and after loading, as shown in Figure 3c. The diffraction peaks indicated that the loaded IL maintained the crystal structure of the pristine SAPO-34, with diffraction peaks at 2θ of 9.56, 12.98, 16.16, 20.76, 26.08, and 31.22° attached to (101), (110), (021), (121), (220), and (401) planes of SAPO-34, respectively.^33,34^ The peaks are of high intensity, indicating that the loaded IL remains well crystalline and suggesting that the IL deposit does not alter the crystal structure of original SAPO-34.

The morphology and particle size of SAPO-34 were characterized by using SEM, as shown in Figure 3d. The commercial SAPO-34 particles display a regular cube and a neatly aligned arrangement, possibly reflecting a CHA structure. The particle size of SAPO-34 was evaluated using the Nano Measurer 1.2 software by randomly selecting 50 crystals for size assessment. The particle sizes are mainly in the range of 4–6 μm.

**Figure 4:**
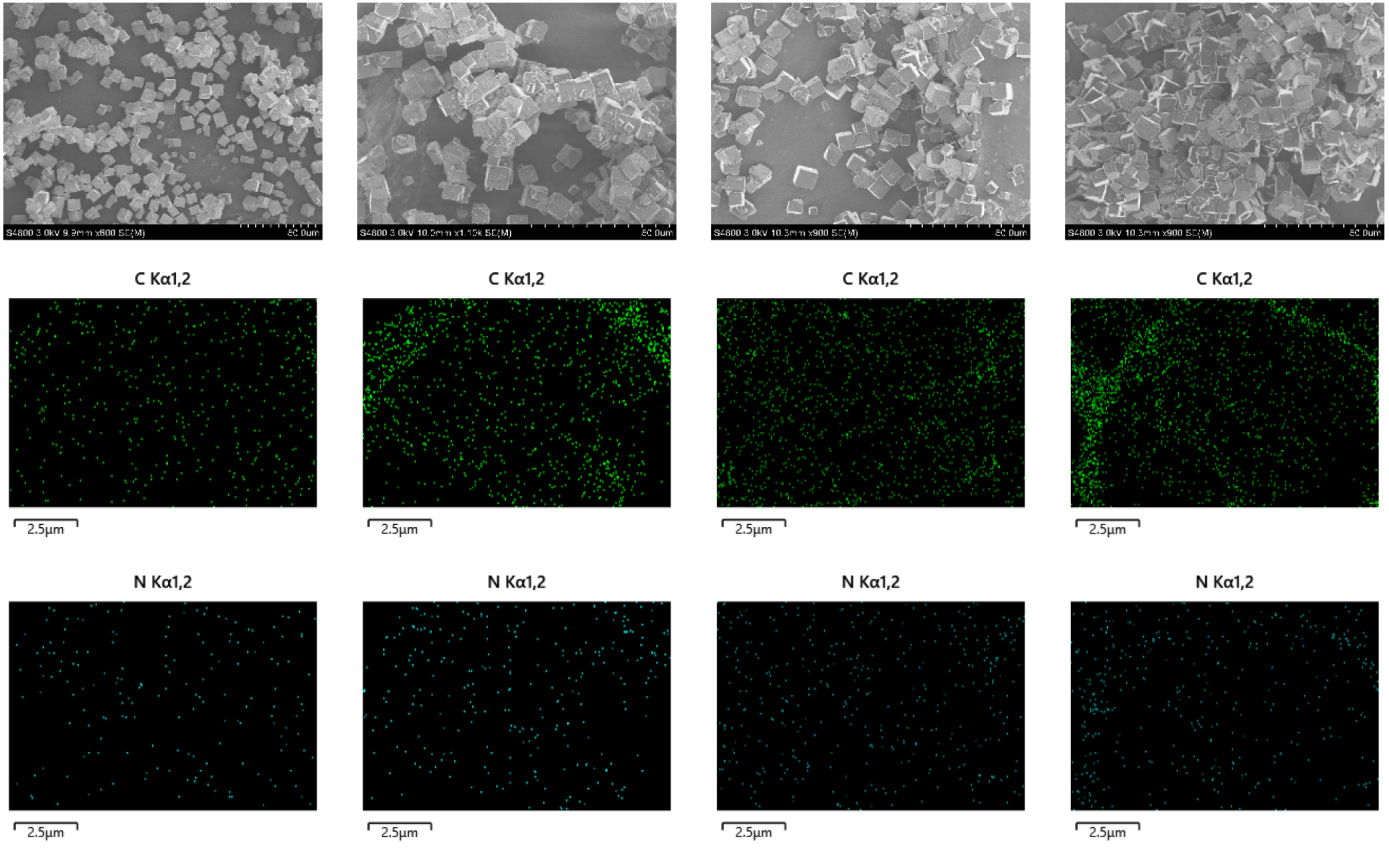
SEM and EDX mapping images of SAPO-34 with varying IL loadings.

The SEM and EDX images in Figure 4 display the analysis of the morphology and surface elements of the composites when different amounts of ILs were loaded onto the surfaces of molecular sieves. It is evident that the loaded samples begin to agglomerate compared to the well-dispersed state of the pristine SAPO-34. As IL loading amount increases, the sample experiences more severe agglomeration buildup. This indicates that the IL on the surface of molecular sieves promotes the agglomeration buildup of SAPO-34. This result is consistent with the results of argon adsorption–desorption characterization. The analysis of the carbon (C) and nitrogen (N) elements associated with the IL on the surface of SAPO-34 indicates that both elements are evenly distributed on the surface of the molecular sieves at low loading amounts. As the IL loading increased, the carbon and nitrogen elements on the surface of the molecular sieve showed a localized increase in distribution density. This suggests that the IL was becoming unevenly distributed on the surface of the sieve and further pile-up.

**Figure 5:**
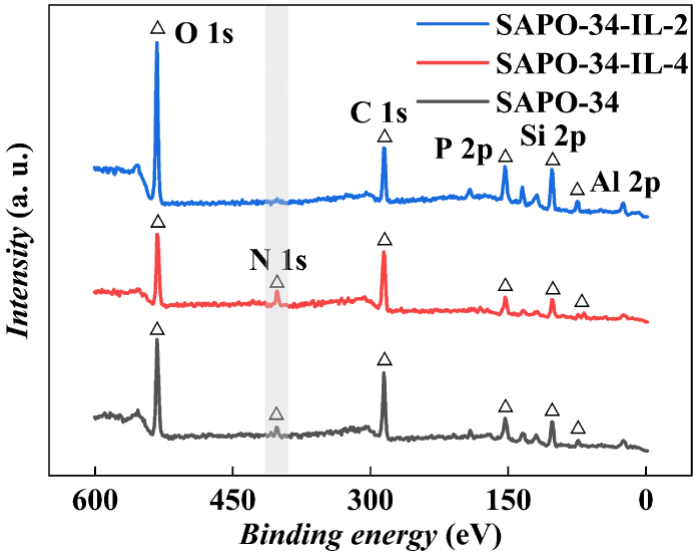
XPS wide scan spectra of SAPO-34, SAPO-34-IL-1, and SAPO-34-IL-2.

The chemical surface compositions of the pristine SAPO-34 and loaded sample SAPO-34-IL were analyzed by using XPS, as depicted in Figure 5. The wide scan profiles of the molecular sieves and loaded samples reveal the elements C, N, oxygen (O), silica (Si), aluminum (Al), and phosphorus (P). The characteristic peaks are clearly visible at 74.4 eV (Al 2p), 102.1 eV (Si 2p), 134.1 eV (P 2p), 284.7 eV (C 1s), 401.7 eV (N 1s), and 532.2 eV (O 1s). The presence of the characteristic peak of the N element, belonging to the IL, in the loaded samples indicates that the IL was successfully loaded onto the molecular sieves. Table 3 shows the atomic percentages of elements on the surface of the molecular sieve, calculated from XPS spectra, for the sample of pristine SAPO-34 and those with two different loading amounts selected for the study. The elemental contents of O, P, Al, and Si on the surface of the pristine SAPO-34 and loaded samples decrease as the IL loading increases. The values for the pristine SAPO-34 and loaded samples are (28.2, 23.6, 19.1), (4.1, 4.0, 3.0), (8.6, 7.9, 4.6), and (24.7, 15.9, 14.8), respectively.

**Table 3:** Surface Composition of SAPO-34 and SAPO-34-IL from XPS Spectra

	surface composition (at %)					
samples	C	N	O	P	Al	Si
SAPO-34	33.5	0.9	28.2	4.1	8.6	24.7
SAPO-34-IL-2	44.2	4.4	23.6	4.0	7.9	15.9
SAPO-34-IL-4	53.0	5.5	19.1	3.0	4.6	14.8

### Analysis of CO_2_ Sorption/Desorption

**Figure 6:**
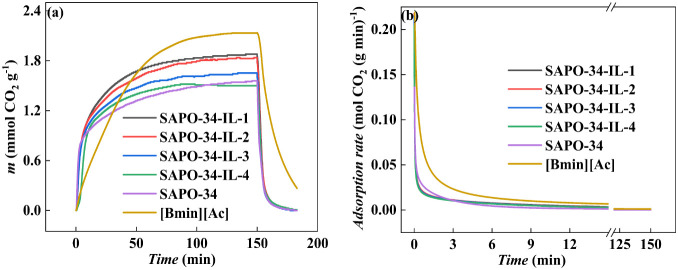
(a) The CO_2_ sorption/desorption and (b) the CO_2_ sorption rate of [Bmim][Ac], SAPO-34-IL, and SAPO-34 based on TGA.

The CO_2_ sorption properties of [Bmim][Ac], pristine SAPO-34, and SAPO-34-IL were conducted using TGA at 303 K, as shown in Figure 6a. The CO_2_ uptakes of pure [Bmim][Ac] and SAPO-34 were 2.132 and 1.558 mmol g^–1^, respectively. The SAPO-34-IL, a composite of IL and molecular sieves, combines the advantages of an absorbent and an adsorbent. The loading amount of IL was 2.84 wt %, and the sample exhibited the highest CO_2_ sorption of 1.879 mmol g^–1^. The CO_2_ sorption decreased as IL loading increased, with CO_2_ sorption amounts being 1.826 mmol g^–1^ (5.40 wt %), 1.652 mmol g^–1^ (9.97 wt %), and 1.499 mmol g^–1^ (15.38 wt %). On the one hand, the IL loaded on the surface of SAPO-34 occupies the outer surface of the molecular sieve,^12,35^ providing stronger sorption sites. On the other hand, due to the small pore size of the molecular sieve, the CO_2_ can enter the pores and diffuse into the adsorption sites of SAPO-34, while the IL remains on the outer surface.^6,36−38^ Therefore, the inner pore structure of the molecular sieve and the IL on its outer surface work together to create sites for CO_2_ capture. Further increasing the loading amount will lead to oversaturation, which blocks the orifice of the molecular sieves and the accumulation of particles. This results in a decrease in the ability to capture CO_2_,^39,40^ which is supported by the argon sorption–desorption and SEM characterization results. Figure 6a,b exhibits the CO_2_ sorption rates of pure [Bmim][Ac], pristine SAPO-34, and SAPO-34-IL. During the first 5 min of CO_2_ sorption, the pure IL exhibited a low sorption capacity and rate, with only 0.281 mmol g^–1^. In contrast, SAPO-34-IL-1 achieved a sorption of 0.847 mmol g^–1^, which is comparable to that of the pristine SAPO-34, and roughly 3 times the sorption of the pure IL. At 10 and 20 min of sorption time, SAPO-34-IL-1, SAPO-34, and IL sorb 1.089 mmol g^–1^ (1.329 mmol g^–1^), 0.951 mmol g^–1^ (1.082 mmol g^–1^), and 0.491 mmol g^–1^ (0.839 mmol g^–1^) of CO_2_, respectively. This represents an increase in sorption of the loaded samples by 14.5% (23.0%) and 121.8% (58.4%) compared to SAPO-34 and IL, respectively. Figure 6b shows the sorption rate versus time. The slope of the pure IL is moderate compared to the pristine SAPO-34 and the loaded samples, due to the high viscosity of IL.^26^ The loaded samples exhibited similar sorption rates. We propose possible sorption processes, in which CO_2_ is initially sorbed rapidly at the sorption sites provided by the IL on the surface of the molecular sieve and then diffuses slowly to the sorption sites in the pore. It was reported that the interaction between the substrate with a high specific surface area and IL resulted in improved dispersion of IL. This resulted in a distinct structure compared to the bulk IL, which in turn enhanced the equilibrium sorption amount and rate.^8,41,42^ As the sorption time progressed, the sorption rates of all of the samples decreased. This is primarily due to the high diffusion resistance of gas in the IL and micropores.

**Figure 7:**
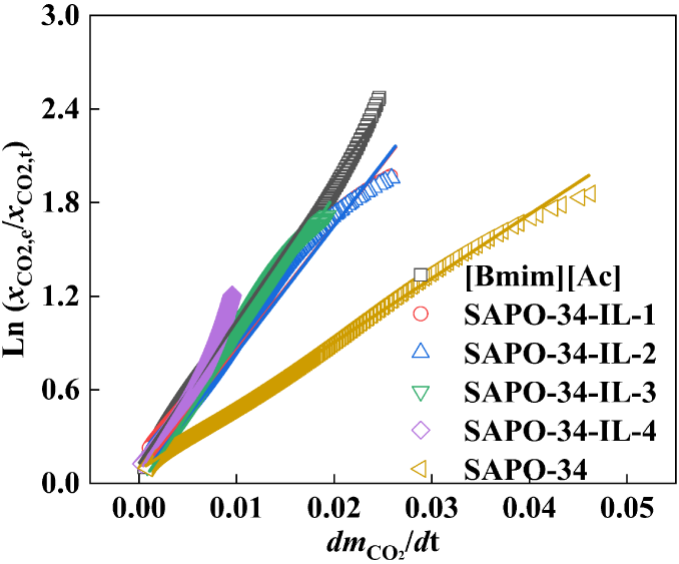
versus  for the CO_2_ uptake of [Bmim][Ac], SAPO-34-IL, and SAPO-34.

According to Figure 6, the significant difference in the sorption rate among bulk IL, pristine SAPO-34, and SAPO-34-IL is primarily due to the diverse apparent mass transfer resistance of CO_2_ sorption in these materials. The rate of CO_2_ sorption, as illustrated in Figure 7, is determined by the apparent chemical potential-based mass transfer resistance, *K*_μ_^–1^, which is calculated using the following equation,^43^ i.e., the ratio of driving force to mass transfer flux.
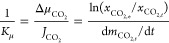
1where  and  represent the mass transfer flux and chemical potential driving force of CO_2_, respectively,  is the amount of sorbed CO_2_ at moment *t*, and  and  are the mole fractions of CO_2_ at the equilibrium moment and at moment *t*, respectively.

Figure 7 shows the apparent mass transfer resistance *K*_μ_^–1^ expressed as the ratio of  and . The slopes of the pure IL and pristine SAPO-34 are 91.37 and 41.03, respectively. This indicates that the mass transfer resistance of CO_2_ for the IL of a certain viscosity hinders the sorption rate, whereas the pristine SAPO-34 sorbs CO_2_ with the smallest resistance. Furthermore, the mass transfer resistances were found to be 81.14 and 82.68 for SAPO-34-IL-1 and SAPO-34-IL-2, respectively. This means that the inclusion of a small amount of IL in SAPO-34 can reduce the mass transfer resistance to some extent compared with the pure IL, thereby enhancing the CO_2_ sorption in the composites. Additionally, the CO_2_ sorption rate also significantly increased compared to pure IL. These indicate that the CO_2_ sorption processes of the composites are simultaneously controlled by the chemical capture of IL, the adsorption of the SAPO-34 together with the mass transfer.^28,41^ Studies have shown that the interaction between the substrate and IL exposed more sorption sites and enhanced the diffusion of CO_2_, improving sorption and kinetics of CO_2_.^25,44^ Further increasing the loading of IL leads to the mass transfer resistances being 93.32 and 102.26 for SAPO-34-IL-3 and SAPO-34-IL-4, respectively, as shown in Table 4. This suggests that the excess IL begins to aggregate and plug the orifices of SAPO-34, which weakens the sorption contribution from IL and molecular sieve. This is consistent with previous studies, which reported that excess IL loading led to a decrease in sorption and rate.^45,46^

**Table 4:** Apparent Chemical-Potential-Based Mass-Transfer Resistance *K*_μ_^–1^ at 303 K

	loading (wt %)	CO_2_ sorption (mmol g^–1^)	*K*_μ_^–1^ sorption	*R*^2^
[Bmim][Ac]	–	2.132	91.37	0.997
SAPO-34-IL-1	2.84	1.879	81.14	0.996
SAPO-34-IL-2	5.40	1.826	82.68	0.997
SAPO-34-IL-3	9.97	1.652	93.32	0.996
SAPO-34-IL-4	15.38	1.499	102.26	0.980
SAPO-34	–	1.558	41.03	0.998

**Figure 8:**
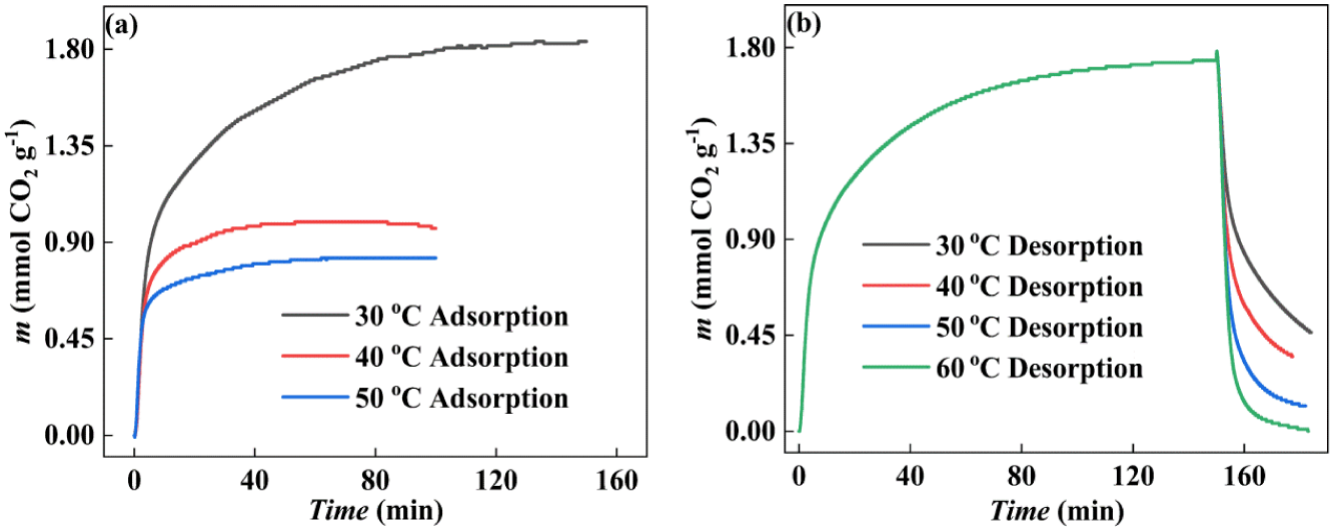
Effect of temperature on CO_2_ sorption/desorption of SAPO-34-IL-2. (a) Sorption and (b) desorption.

Here, we selected SAPO-34-IL-2 to investigate the impact of temperatures on CO_2_ sorption and desorption. Figure 8a shows that as the sorption temperature increases from 303 K to 313 K and 323 K, the CO_2_ sorption amount decreases from 1.826 mmol to 0.995 mmol and 0.827 mmol g^–1^, respectively. This indicates that an increase in the temperature had an adverse effect on the equilibrium sorbed amount of CO_2_. Research has demonstrated that the [Bmim][Ac] reacts with CO_2_, and the acetate anion can replace the acidic proton at the C (2) position of the cation to form an *n*-heterocyclic carbene in a reversible reaction.^47,48^ As a result, the temperature rise caused the positive sorption reaction to convert to the reverse desorption reaction.^49,50^

The desorption process of CO_2_ was investigated at 303, 313, 323, and 333 K under a N_2_ atmosphere. The results are presented in Figure 8b, which clearly shows that the CO_2_ desorption becomes increasingly complete with increasing temperature. After 30 min at 303 K, 26.1% of sorbed CO_2_ remained undesorbed. The desorbed residual CO_2_ was 20.1%, 6.7%, and almost completely desorbed as the temperature was successively increased to 313, 323, and 333 K. These results indicate that [Bmim][Ac] complexed with SAPO-34 has the potential to be an effective sorbent for CO_2_ capture, achieving complete desorption at lower temperatures (333 K).

In this work, the ideal selectivity (*S*) is defined using the simplest definition, i.e., the molar ratio of gas sorption determined by isotherms of pure components at the same pressure,^35^ as the following equation
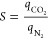
2

Here, *q* is the sorption capacity of the component.

**Figure 9:**
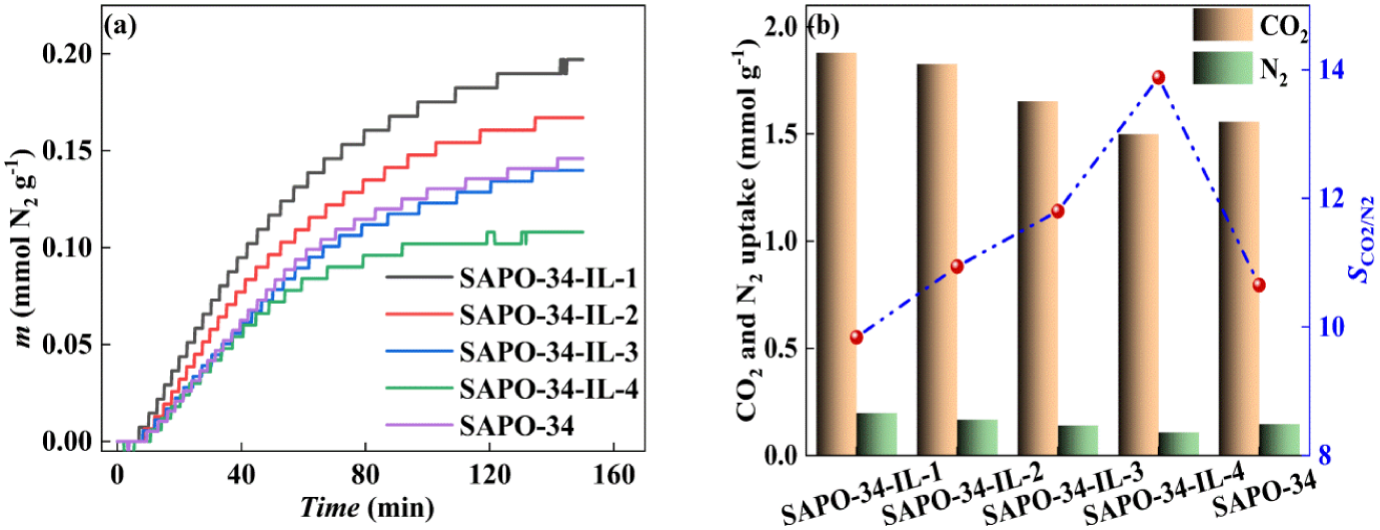
(a) Sorption amount of N_2_ and (b) selectivity of CO_2_/N_2_ for SAPO-34-IL and SAPO-34.

Figure 9a exhibits the N_2_ sorption of SAPO-34 and SAPO-34-IL through TGA at 303 K and 1.0 bar. The sorption curves of the samples under a N_2_ atmosphere exhibit a slow rate. Even after 150 min of sorption time, the sorption equilibrium could not be reached. The pristine SAPO-34 sorbed 1.558 mmol g^–1^ of CO_2_ and 0.146 mmol g^–1^ of N_2_, with an ideal selectivity of 10.65. At a loading of 2.84 wt %, the sample sorbed 1.879 mmol g^–1^ of CO_2_ and 0.197 mmol g^–1^ of N_2_. When the IL loading increases to 5.40, 9.97, and 15.38 wt %, the sorption amount reaches 1.826, 1.652, and 1.499 mmol g^–1^ of CO_2_ and 0.167, 0.140, and 0.108 mmol g^–1^ of N_2_, respectively. Figure 9b displays the selectivity of CO_2_/N_2_ for the pristine SAPO-34 and SAPO-34-IL. Consequently, their selectivity values are 9.84, 10.94, 11.80, and 13.88 for different samples; i.e., the selectivity of SAPO-34-IL increases as the mass ratio of IL to SAPO-34 increases. The composite samples exhibited a decreasing trend in the sorptions of CO_2_ and N_2_ by increasing the loading of IL. The presence of IL leads to an increased selectivity of the studied composites for CO_2_, which is consistent with the findings reported in the literature.^51−53^ Related literature reported that the coverage of the pores by a small amount of IL results in an increased surface polarity and a decreased hydrophobicity of SAPO-34. This, in turn, impedes N_2_ sorption while promoting the sorption and selectivity of CO_2_ by the SAPO-34-IL.^12^

**Figure 10:**
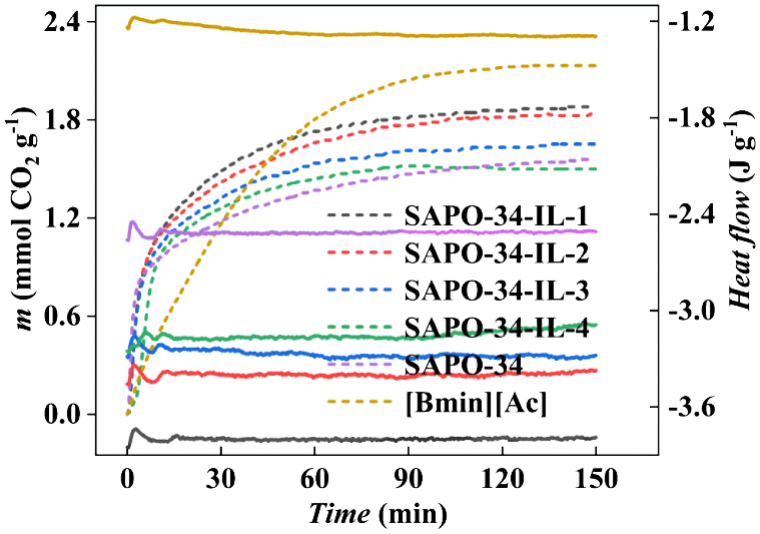
Heat flow of CO_2_ sorption as a function of time depended on the DSC.

The interaction strength between the CO_2_ molecules and the sorbent was determined using DSC. The results are shown in Figure 10. The CO_2_ desorption heat was determined based on the heat released during CO_2_ sorption and the heat required for CO_2_ sorption.^54,55^ The sample is allowed to sorb CO_2_ to equilibrium under a CO_2_ atmosphere at 303 K. The heat flow curves from the sorption stage were used to calculate the heat required to uptake one mole of CO_2_. The results revealed that the CO_2_ desorption heat for the pristine SAPO-34 is 29.3 kJ mol^–1^, which is consistent with values reported in the literature (30 kJ mol^–1^, 33 kJ mol^–1^, and 34 kJ mol^–1^).^15,56^ This is because of the surface properties of the adsorbent, which can regulate the interaction between CO_2_ and the adsorbent.^57^ Furthermore, the heat required for CO_2_ desorption from the IL [Bmim][Ac] was found to be 43.0 kJ mol^–1^, a value similar to 38 kJ mol^–1^ reported in the literature.^58,59^ Also, the values obtained were 30.6, 33.5, 38.9, and 40.8 kJ mol^–1^ for SAPO-34-IL with 2.84, 5.40, 9.97, and 15.38 wt % loading amount, respectively. It can be seen that the CO_2_ desorption heat increases with an increase in IL loading. The CO_2_ desorption heat in the loaded samples was intermediate between that of pristine SAPO-34 and pure IL, which is mainly due to the interaction between CO_2_ and IL as well as SAPO-34.

Generally, an ideal sorbent should possess high CO_2_ sorption capacity and low desorption heat.^60^ Therefore, we conducted a comprehensive comparison of the studied systems, including the method, sorption/desorption operating conditions, sample mass, CO_2_ sorption capacity (*m*), and desorption heat (*Q*) reported in the literature with those conducted in this work. The findings are summarized in Table 5, showing that supported ILs with lower CO_2_ desorption heat (or higher desorption heat) usually exhibit lower CO_2_ sorption capacity (or higher CO_2_ sorption amount), such as ZIF-8-[N_2114_][Ac],^35^ HEG-[Bmim][BF_4_],^61^ MWNTs-[Vmim][BF_4_],^62^ and SBA15-[TEPA][NO_3_].^25^ The desorptions were usually performed under vacuum conditions or above 363 K.^63^ Furthermore, we selected the typical physical absorption method using water as a solvent, which has low CO_2_ desorption heat, and the chemical absorption method using a 30 wt % MEA solution, which has a high CO_2_ uptake rate and uptake as benchmarks at 303 K and 1.0 bar. To ensure precision, we compared the CO_2_ sorption and desorption heats of IL-loaded substrate sorbents at 303 K (or 298 K) and 1.0 bar reported in the literature with our work. The sorption and CO_2_ desorption heat of SAPO-34-IL with IL loading of 2.84 wt % are 1.879 mmol g^–1^ and 30.6 kJ mol^–1^, respectively, which are superior to the results reported in the literature at the same pressure and same or similar temperature. When the IL loading is increased, the corresponding sorption and desorption heats are 1.826 mmol g^–1^ (33.5 kJ mol^–1^), 1.652 mmol g^–1^ (38.9 kJ mol^–1^), and 1.499 mmol g^–1^ (40.8 kJ mol^–1^), respectively. The results show that the samples in our work with varying IL loading are located in the upper-left region of the figure, possessing the advantages of high sorption and low desorption heat. The sample prepared in this work combines the advantages of physical and chemical sorption methods and has significant potential for future CO_2_ capture.

**Table 5:** Comparison of the Supported IL for Capturing CO_2_ in the Literature with this Work

supported-ionic liquid	sorption (technique, sorption conditions, sample mass)	*m* (mmol g^–1^)	*Q* (kJ mol^–1^)	desorption	refs
HKUST-[Bmim][Ac]	aIGA, CO_2_ at 303 K, 0.15 bar, 80 mg	1.7	∼48	N_2_ at 373 K, 50 mL min^–1^	(64)
HEG-[DAMT][BF_4_]	bVSA, CO_2_ at 298 K, 1.0 bar, 150–200 mg	0.557	17 ± 2	–	(65)
UiO-67-IL (1)	cVGS, CO_2_ at 298 K, 1.0 bar, ∼200 mg	1.02	26 ± 1	vacuum at 363 K	(63)
ZIF-8-[N_12_OH_2_OH_2_OH][Ac]	dMSB, CO_2_ at 303 K, 1.0 bar	0.57	12.60	vacuum at 373 K	(35)
ZIF-8-[N_2114_][Ac]	dMSB, CO_2_ at 303 K, 1.0 bar	0.56	12.10	vacuum at 373 K	
[Cho][Arg]/Cu-MOF	eQCM sensor, CO_2_ at 303 K, 1.0 bar	0.219	50.3	–	(66)
[Cho][Pro]/Cu-MOF	eQCM sensor, CO_2_ at 303 K, 1.0 bar	0.208	48.9	–	
[Cho][Tyr]/Cu-MOF	eQCM sensor, CO_2_ at 303 K, 1.0 bar	0.198	35.1	–	
SBA15-[TEPA][NO_3_]	fBT, 15% CO_2_/N_2_ at 333 K, 0.15 bar, 500 mg	2.15	64.00	–	(25)
ZIF-8-[Emim][Gly]	aIGA, CO_2_ at 303 K, 0.2 bar, 50–70 mg	0.89	∼59	N_2_ at 373 K, 100 mL min^–1^	(67)
ZIF-8-[Emim][Ala]	aIGA, CO_2_ at 303 K, 0.2 bar, 50–70 mg	0.91	∼55	N_2_ at 373 K, 100 mL min^–1^	
OMS-IL(Lys)	gTGA, 50% CO_2_/N_2_ at 298 K, 1.0 bar, 20 mg	0.61	85.7	N_2_ at 378 K, 50 mL min^–1^	(54)
OMS-IL(Pro)	gTGA, 50% CO_2_/N_2_ at 298 K, 1.0 bar, 20 mg	0.53	85.15	N_2_ at 378 K, 50 mL min^–1^	
OMS-IL(Gly)	gTGA, 50% CO_2_/N_2_ at 298 K, 1.0 bar, 20 mg	0.50	76.14	N_2_ at 378 K, 50 mL min^–1^	
OMS-IL(Ala)	TGA,^g^ 50% CO_2_/N_2_ at 298 K, 1.0 bar, 20 mg	0.50	67.72	N_2_ at 378 K, 50 mL min^–1^	
HEG–[Bmim][BF_4_]	hPSA, CO_2_ at 298 K, 1.0 bar, 200 mg	0.512	21 ± 1	reduced pressures	(61)
HEG–poly([Vmim][BF_4_])	hPSA, CO_2_ at 298 K, 1.0 bar, 200 mg	0.618	23.5 ± 0.8	reduced pressures	
MWNTs-[Vmim][BF_4_]	hPSA, CO_2_ at 283 K, 1.0 bar, 150–200 mg	0.11	15 ± 1	reduced pressures	(62)
MWNTs- poly([Vmim][BF_4_])	hPSA, CO_2_ at 283 K, 1.0 bar, 150–200 mg	0.137	27 ± 2	reduced pressures	
PEMWNTs-[VMIM][BF_4_]	hPSA, CO_2_ at 283 K, 1.0 bar, 150–200 mg	0.410	23 ± 2	reduced pressures	
PEMWNTs- poly([Vmim][BF_4_])	hPSA, CO_2_ at 283 K, 1.0 bar, 150–200 mg	0.491	30 ± 3	reduced pressures	
30% MEA	–	2.749	85.6	–	(68,69)
H_2_O	–	0.032	17.34	–	(70)
SAPO-34-[Bmim][Ac]	gTGA, 50% CO_2_/N_2_ at 303 K, 1.0 bar, 2–3 mg	1.879	30.6	N_2_ at 333 K	this work

a IGA: intelligent gravimetric analyzer.

b VSA: vacuum-swing adsorption technique.

c VGS: volumetric gas sorption instrument.

d MSB: magnetic-suspension balance.

e QCM sensor: the cell of adsorption entails an 8 MHz AT-cut quartz crystal applied in the electrical oscillator circuit.

f BT: breakthrough experiments.

g TGA: thermogravimetric analyses.

h PSA: pressure-swing adsorption method.

**Figure 11:**
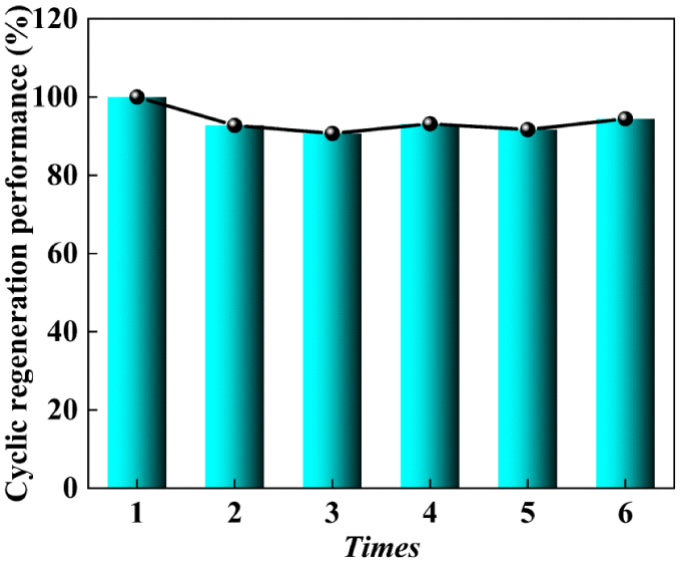
Cyclic regeneration of CO_2_ in SAPO-34-IL at 303 K.

The cyclic regeneration performance of CO_2_ sorption/desorption sorbent is an important index for evaluating sorbents. The ideal sorbents can maintain a stable sorption performance after a certain number of cyclic regenerations. According to the cycling experiments reported in the literature, the desorption cycling test was carried out at 333 K, as shown in Figure 11. The experimental results clearly demonstrate that after the second cycle, the sorption efficiency is 92.7%, and after the sixth cycle, the sorption efficiency is 94.5%, and the sorption efficiency of CO_2_ from the second to the sixth cycle is basically unchanged. These results indicate that SAPO-34-IL has excellent cycling stability and is a promising CO_2_ sorbent.

## Conclusions

In conclusion, we used the impregnation method to complex molecular sieve SAPO-34 with IL [Bmim][Ac] for CO_2_ capture. The characterization results showed that the loading of IL did not alter the crystal structure of the pristine SAPO-34. Furthermore, IL was found to be primarily distributed on the surface of the molecular sieve. TGA was used to study the sorption of CO_2_ on SAPO-34-IL. The results showed that the loaded samples, coupled with molecular sieves and IL, sorbed CO_2_ up to 1.879 mmol g^–1^ at 303 K and 1.0 bar. The sorption rate was higher than that of pure IL, indicating that the composites strengthened the equilibrium sorption amount and rate. The resistance analysis revealed that the CO_2_ sorption of loaded samples was 11.2% lower than that of the pure IL. Additionally, the IL-loaded molecular sieves improved the selectivity of CO_2_/N_2_. The DSC results showed that the CO_2_ desorption heat for the loaded samples was moderate, with values ranging from 30.6 to 40.8 kJ mol^–1^, which possesses high sorption and low desorption heat. This work contributes to the future development of IL-loaded molecular sieve materials for CO_2_ capture.
